# Analysis of trends and variability of hydroclimate variables in the Didessa Sub-basin: Implications for the Upper Blue Nile (Abbay) Basin, Ethiopia

**DOI:** 10.1016/j.heliyon.2024.e40530

**Published:** 2024-11-22

**Authors:** Selamawit Bekele Degefu, Tena Alamirew, Sintayehu Fetene Demessie

**Affiliations:** a Ethiopian Institute of Water Resources, Addis Ababa University, Ethiopia; b Ethiopian Institute of Agricultural Research, Ethiopia; c College of Business, Technology and Vocational Education, Kotebe University of Education, Addis Ababa, Ethiopia; d Africa Center of Excellence for Climate-Smart Agriculture and Biodiversity Conservation, Haramaya University, Ethiopia

**Keywords:** Didessa sub-basin, Hydroclimate, Modified Mann-Kendall, Standardized anomaly index, Sustainable water management, Trend analysis

## Abstract

Long-term trends and variability of hydroclimate variables significantly impact water resources. This study aims to investigate trends and variability of hydroclimate variables in the Didessa sub-basin. Modified Mann-Kendall and Sen's slope estimators were used to analyze the trends. At the same time, variability was assessed using the standardized anomaly index (SAI), precipitation concentration index (PCI), and coefficient of variation (CV). The seasonal and annual rainfall trend analysis results showed an increasing tendency, except for the winter. The PCI and SAI results for most of the stations in the sub-basin exhibit moderate rainfall concentration. The sub-basin mean temperature showed a significant upward trend, with annual average rates of +0.02 °C. Analysis of streamflow at Arjo and Bunobedele stations showed a significant upward trend of 0.5 m^3^/s/yr and 0.015 m^3^/s/yr, respectively, in the spring season. Mixed trends were observed at upstream stations; Big Anger exhibits a downward trend with a declining rate of 3.04 m^3^/s/yr, while Little Anger displays a rising trend with an increasing rate of 0.25 m^3^/s/yr. Flow data at upstream and downstream gauged stations positively correlated with mean rainfall. The findings of this study have significant implications for water resource management and climate change mitigation by giving insight to policymakers and water resource managers in developing adaptation strategies for changing hydroclimate conditions to ensure sustainable water utilization globally.

## 1 Introduction

Climate change is one of the twenty-first century's most significant challenges and a recognized grave concern. The likelihood of climate change is now widely acknowledged, necessitating certain adjustments [1]. Climatic and hydrological variable fluctuations and their correlations are typically used to characterize climate variability and changes [2,3]. Factors such as changes in solar energy, volcanic eruptions, fluxes of atmospheric concentrations of greenhouse gases (GHGs) like CO2, and shifts in land cover determine these fluctuations and changes [4]. Both human activities and natural climatic and terrestrial processes can alter these variables. There is strong evidence that human activity has caused significant changes in the terrestrial climate and natural environment over the past century [5]. Therefore, identifying long-term trends in hydroclimatic variables and understanding past and present climate changes is crucial in climate change studies [2,6].

Temperature and rainfall are the two closely related variables in climate change research. Their deviation from the long term is the benchmark in climate change studies. Consequently, most studies on climate change emphasize trend analysis of these variables. These factors affect the frequency of waterfalls via the hydrological cycle, affecting the amount of water available [3,7,8]. Polar ice caps have melted as temperatures rise, increasing evapotranspiration and average sea levels while decreasing infiltration, freshwater availability, and soil humidity [9]. Temperature changes impact rainfall, and as air temperatures rise, the amount and distribution of rainfall patterns also changed [10]. Variations in temperature, rainfall, and other climatic factors affect streamflow levels and the timing of hydrologic events. These directly impact water availability for different sectors, agricultural productivity, and ecosystems and alter the patterns of hydrological disasters such as floods and droughts [1,11,12]. Variability and changes in precipitation and temperature will significantly impact agricultural and water management practices, particularly in countries like Ethiopia, which rely heavily on rain-fed agriculture [13].

According to the Intergovernmental Panel on Climate Change (IPCC), human activity has caused worldwide warming, with the global surface temperature rising by 1.1 °C between 2011 and 2020, primarily due to greenhouse gas emissions. The ongoing rise in global greenhouse gas emissions has been attributed to unequal historical and contemporary contributions from unsustainable energy usage, land use change, consumption habits and lifestyles, and production within and across nations and among individuals. The average global land surface temperature will increase over the 21st century. The likely global increment in temperature during 1900 compared to 1850 is + 1.09 °C, and there is an increment of +0.99 °C from 2001 to 2020 over the period of 1850–1900. This indicates the earth is experiencing the most rapid warming ever recorded [14].

Hydroclimate trend and variability analysis examine long-term changes in key hydrological and climatic variables such as rainfall, temperature, and streamflow. These trends provide valuable insights into how the climate evolves and its implications for water availability, ecosystems, and socio-economic activities [15]. Various studies have been conducted on hydroclimate variable trends and variability in the Abbay River basin. Research conducted in the Abbay basin on hydroclimate data subjected to trend analysis tests revealed a significant increase in temperature on a seasonal and annual time scale, along with fluctuating trends in seasonal and extreme streamflow. However, no significant variations were observed in precipitation on either a seasonal or annual time scale [16]. A study using the Mann-Kendall analysis on rainfall and streamflow data in the Abbay Basin observed an increasing trend in streamflow during the rainy season but a decrease during the dry season. However, no significant trends were observed in total the seasonal and annual rainfall over the basin [17].

Researchers found a statistically significant increase in temperature on both seasonal and annual time scales at several locations in Ethiopia, indicating a rising trend in temperature [1,2,[18], [19], [20]]. A study assessing rainfall trends in the Abbay basin revealed irregular patterns, with a non-significant mix of positive and negative trends observed at annual and seasonal time scales [8,21]. Asfaw et al. and Dechassa et al. observed high rainfall variability in the Abbay River basin for the Woleka and Didessa sub-basins at monthly and seasonal scale [20,22]. Research findings on hydroclimate variables in the Birr River watershed outlined a substantial increasing and decreasing trend for stream flow data with a non-significant trend in precipitation [15].

Trend analysis of hydroclimate variables is vital for effective water resource management, as understanding the temporal and spatial variability in these variables enables more accurate estimates of climate change, guiding policy decisions. However, there is a lack of detailed studies on hydroclimate variable trends in western Ethiopia, especially in the Didessa sub-basin. Most existing research in Ethiopia focuses primarily on precipitation and temperature trends using the classical Mann-Kendall (MK) test. Still, it fails to explore the interactions between hydrology and climate, such as the relationship between streamflow and precipitation. This gap highlights the need for comprehensive studies that analyze the interplay between rainfall and streamflow, offering insights into water management strategies.

This study addresses that need by examining the spatiotemporal trends and variability of hydroclimate variables in the Didessa sub-basin, emphasizing the implications of climate change on water resources and related sectors. It employs a comprehensive approach, using a combination of the Modified Mann-Kendall test, Sen's slope estimator, Standardized Anomaly Index (SAI), Precipitation Concentration Index (PCI), and Coefficient of Variation (CV). Unlike previous studies focusing on a single method or variable, this research provides a multifaceted analysis, offering a deeper understanding of upstream and downstream stations' trends and spatial differences in streamflow. By correlating streamflow data with mean rainfall and identifying seasonal trends, this study provides actionable insights for water resource management and climate change mitigation in the basins where rapid hydro-infrastructure is under development, which has implications for transboundary water resource management.

The Didessa River, found in the Didessa sub-basin, is the main tributary of the Abbay River, contributing approximately one-fourth of the river's total flow, making it crucial in terms of water volume. This high contribution to the Abbay River's flow underscores the importance of understanding the hydroclimate trends and variability in the Didessa sub-basin. Any changes in the sub-basin can significantly impact the water availability and management in the entire Abbay River, making it a key area for climate change research and water resource management [23].

Despite its crucial role in the Abbay River and the development of major water resource projects, the Didessa sub-basin has yet to be thoroughly investigated regarding the variability and trends of climate variables and their impact on streamflow, unlike the other sub-basins. This study, therefore, aimed to fill this gap by investigating the spatiotemporal trend and variability of hydroclimate variables in the Didessa sub-basin at monthly, seasonal, and annual time scales, highlighting this region's significance in global climate change research.

## 2 Methods

### 2.1 Description of the study area

**Fig. 1:**
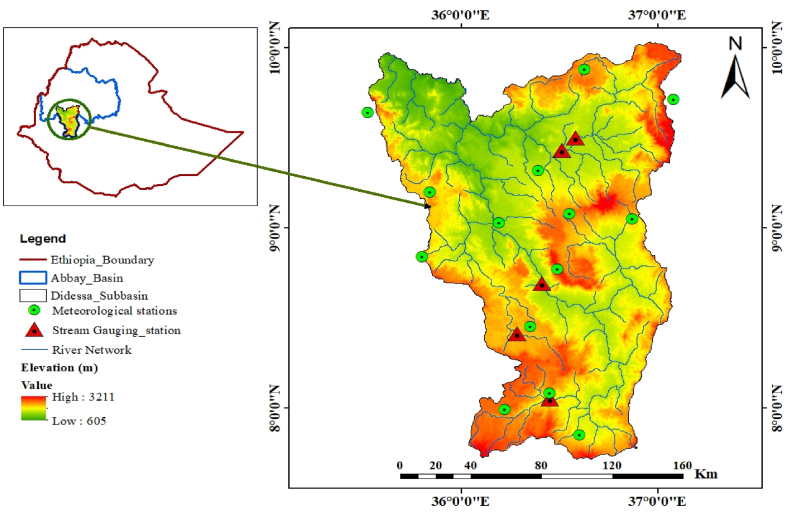
Location map of Hydrological and Meteorological stations in the study area.

Didessa sub-basin is found in the southwestern part of the Abbay River Basin. Geographically, it is situated between the latitudes of 7°42′43″N to 10°2′55″N and the longitude of 35°31′23″E to 37°7′60″E. Didessa sub-basin has a highly varied topography, ranging from 605 to 3211 m above mean sea level, as described in (Fig. 1). Based on Ethiopian climate classification by elevation, the sub-basin is classified into three agroecological zones, Dega, Woyna-dega, and Kola, with respective percentile coverage of 7 %, 45.8 %, and 47 % [22]. The Didessa River is Ethiopia's largest Abbay River tributary, with a catchment area of 28,175 km^2^. Its volume of water contributes around one-fourth of the Abbay River's total flow [23,24]. The average daily maximum and minimum temperatures in the sub-basin are 26.5 °C and 13 °C, respectively. The sub-basin receives an average of 1750 mm annual rainfall, of which 70 % occurs from June to September (summer season). The sub-basin soil is often deep and rich in organic matter, indicating a significant potential for infiltration. Acrisols are the area's most common form of soil; however, nitisols and cambisols are also frequently found. The primary land cover types include forest, shrubland, grassland, and agricultural land, with rainfed agriculture being the common farming practice in the area [24].

### 2.2 Hydroclimate data Collection

Daily climate data on rainfall and maximum and minimum temperatures were obtained from the Ethiopian Meteorological Institute (EMI). Stations with over 30 years of data were considered to ensure data reliability for application to climate change trend analysis [25]. For this study, 42 years of relatively continuous and high-quality climate data, covering the period from 1981 to 2022, were considered for climate data analysis. Fourteen stations representing the study area were selected based on the availability of quality data, with less than 10 % missing (Table 1).

**Table 1:** Description of the meteorological station and database period.

Stations	Geographical Location			Database period (year)
	Latitude	Longitude	Altitude (m)	
Agaro	7.85	36.60	1465	1981–2022
Anger	9.32	36.39	2467	1981–2022
Arjo	8.77	36.49	1287	1981–2022
Bedele	8.45	36.35	2361	1981–2022
Dembi	8.08	36.45	1877	1981–2022
Didessa	9.03	36.19	1287	1981–2022
Gatira	7.99	36.22	2460	1981–2022
Gidayana	9.88	36.63	1826	1981–2022
Gimbi	9.20	35.84	1800	1981–2022
Nedjo	9.64	35.52	1385	1981–2022
Nekemte	9.08	36.55	2119	1981–2022
Nolekaba	8.84	35.80	1844	1981–2022
Shambu	9.72	37.08	2000	1981–2022
Sibu sire	9.05	36.87	1939	1981–2020

Daily streamflow data from five gauging stations were acquired from the Ethiopia Ministry of Water and Energy (MoWE) (Table 2). Based on flow data availability, three stations from downstream and two from upstream were chosen with different recording years. Limited data is available in the upstream gaged stations due to their long-term lack of proper functionality.

**Table 2:** Description of selected gaged stations and database period.

River	Station	Geographical Location		Catchment area (km^2^)	Database period (Year)
		Latitude	Longitude		
Didessa	Dembi	9.05	36.58	1806	1987–2016
Dabana	Bunobedele	8.40	36.29	47	1984–2016
Didessa	Arjo	8.68	36.42	9981	1981–2016
Anger	Big Anger	9.43	36.51	4674	1994–2004
Anger	Little Anger	9.50	36.58	844	1981–2002

**Fig. 2:**
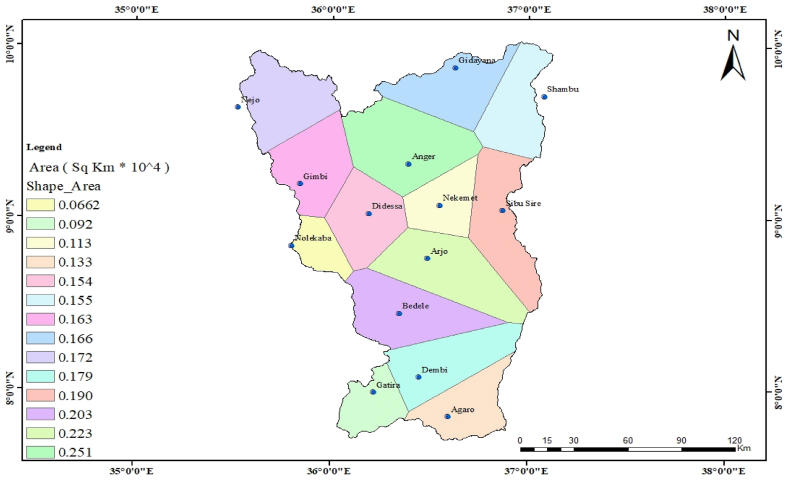
Station's area coverage.

**Weighted average rainfall:** The weighted rainfall for the sub-basin was calculated by multiplying each station's total rainfall by the area of its corresponding polygon, as described in (Fig. 2). The sum of these products was then divided by the total area of the sub-basin to obtain the final weighted average rainfall (equations (1), (2))). The area coverage of the polygon for each station was determined using Thiessen's polygon method, which is a widely accepted technique in ArcGIS [26].

(1) $$Pav=\frac{P_{1}A_{1}+P_{2}A_{2}+P_{3}A_{3}+.\;.\;.+P_{n}A_{n}}{A_{1}+A_{2}+A_{3}+.\;.\;.+A_{n}}$$

(2) $$Pav=\sum_{i=1}^{m}Pi\frac{A_{i}}{A}$$

Where m is the number of stations, and the ratio Ai/A is called the weighting factor for each station.

### 2.3 Data quality control and gap Filling

The hydro-climate variables of the sub-basin were tested for homogeneity and autocorrelation. The homogeneity and autocorrelation of the hydro climate data were assessed using Pettit's test in the XLSTAT tool and the Autocorrelation function using (ACF) package in R software, respectively. Throughout the quality control process, days with a maximum temperature lower than the minimum temperature and precipitation with negative readings were investigated and corrected using data from neighboring stations. Additionally, data points that deviated more than four times from the standard deviation were considered outliers and were replaced with the mean values of the day before and after the outlier. Missing climate data were estimated using multiple imputation methods in Excel-XLSTAT Add-ins [27].

### 2.4 Data analysis

#### 2.4.1 Temporal and spatial trend analysis of hydroclimatic variables

The data analysis aimed to identify, calculate, and confirm the variability and trends in the sub-basin's hydroclimate variables. Different trend and variability analysis methods were used to detect the trends and variability of these variables. For the analysis, daily hydroclimate data was aggregated into monthly time series, which were further used to generate seasonal and annual time series data.

##### 2.4.1.1 Modified Mann-Kendall test and Sen's slope

The Modified Mann-Kendall trend test was used to detect trends in time series data for the hydroclimatic variables. This test is often paired with Sen's Slope Estimator to quantify the trend's magnitude by calculating the median slope between all data points, providing a robust estimate of the rate of change.

The Modified Mann–Kendall trend test associated with the Sen's slope is a widely used non-parametric trend analysis method in hydroclimate research to detect trends of hydroclimate variables. This nonparametric analysis method is particularly effective in identifying monotonic trends in hydroclimate and related data. The nonparametric MK trend test has a significant advantage over the parametric test, as it is better suited for data with missing values, non-normal distribution, and outliers [28]. This advantage has led to the widespread use of the Mann–Kendall trend test for trend analysis for hydroclimate variables by different researchers [20,[29], [30], [31]].

The Mann-Kendall test runs based on two hypotheses: the null hypothesis, which states that there is no trend in the data series and that it is serially independent and identically distributed, and the alternate hypothesis, which states a trend in the data series. All trend data have been assessed at the 0.05 significance level to evaluate the study area's trend characteristics accurately. The Mann-Kendall test rejects the null hypothesis of no trend when the calculated |Z| > 1.96 at a 5 % confidence level (Equation (6)). The Z and S statistics indicate trends, with positive values representing increasing trends and negative values representing decreasing trends. The Z value and computed p-value are compared to the user-defined confidence level of the normal distribution to assess the trend.

The MK test statistic 'S' is a key component in the test. It increases by 1 when a data value from a later period exceeds a previously sampled data value. Conversely, if the data set shows the opposite trend, the 'S' value decreases by 1. The sum of these increments and decrements determines the final value of 'S.' The MK test statistic 'S' is computed using the formula described [32] (Equation (3)).

(3) $$S=\sum_{i=1}^{n-1}\sum_{k=i+1}^{n}sgn\left(x_{k}-x_{i}\right)$$

The trend test is applied to a time series Xi, ranked from i = 1, 2 … n-1, and x_k_, ranked from k = i + 1, 2 … n. x_i_ is a reference point for each data point compared with the rest of the data points x_k._ Sgn (xk-xi) is a sign function that detects the direction of change (upward, downward, or no change) between two data points over time, as described in (equation 4).

(4) $$sgn\left(x_{k}-x_{i}\right)=\left\{ \begin{array}{c} +1\;if\;\left(x_{k}-x_{i}\right)>0\;\text{Posetive}\;\text{trend}\\ 0\;if\;\left(x_{k}-x_{i}\right)=0\;\text{No}\;\text{change}\\ -1\;if\;\left(x_{k}-x_{i}\right)\langle 0\;\text{Negative}\;\text{trend}< \end{array}\right.$$

Where x_i_ and x_k_ are the annual values in years i and k (k > i).

It has been documented that when the number of observations is more than 10 (n > 10), the statistic 'S' is approximately normally distributed with the mean, and S becomes 0 [33]. In this case, the variance statistic is given as (Equation (5))

(5) $$Var\left(S\right)=\frac{\left[n\left(n-1\right)\left(2n+5\right)-\sum_{t}t\left(t-1\right)\left(2t+5\right)\right]}{18}$$

Where n is the number of observations and ti are the ties of the sample time series.

Finally, the test statistics Z is as computed as follows (Equation (6))

(6) $$`\;Z=\left\{ \begin{array}{c} \left(S-1\right)/\sqrt{Var\left(S\right)}\;if\;S>0\\ \;0\;if\;S=0\\ \left(S+1\right)/\sqrt{Var\left(S\right)}\;if\;S\langle 0< \end{array}\right.$$

where S = any integer between $-n\left(\frac{\left(n-1\right)}{2}\right)\;and\;n\left(\frac{\left(n-1\right)}{2}\right)$ xk and xi are sequential data values, n is the length of the data set, the notation t is the extent of any given time, and Rt denotes the summation of all relationships.

**Sen's slope (β)** estimates the rate of change per unit of time in a trend that is not impacted by outliers or extreme values in the data [34]. As a result, it is a highly accurate and reliable tool for developing linear relationships instead of using regression slopes. Sen's slope was calculated as per the procedure described by Ref. [34] (Equation (7)),

(7) $$\beta =Median\;\left(\frac{x_{i-}x_{k}}{i-k}\right)$$

In which 1 < k < i < n. The estimator β is the median over all combinations of record pairs for the whole data set. A positive value indicates an 'upward trend,' and a negative value indicates a 'downward trend.'

Serial autocorrelation can increase the likelihood of the Modified Mann-Kendall (MMK) test detecting a significant trend. To ensure the data's suitability for the MMK test, it is important to use serially independent or uncorrelated data. In this study, lag-1 autocorrelation was evaluated to mitigate autocorrelation in hydroclimate data. Homogeneity and autocorrelation were assessed using Pettitt's test in the XLSTAT tool and the Autocorrelation Function (ACF) in R software. The ACF was used to identify cross-correlation or cross-covariance in the time series data. To address lag-1 autocorrelation, the Bias Corrected Pre-whitening (bcpw) package in R was employed to pre-whiten the data, thereby removing biases as recommended [35].

#### 2.4.2 Temporal and spatial variability analysis of hydroclimatic variables

This variability of hydroclimatic variables was analyzed using descriptive statistics such as the Coefficient of Variation (CV), Standard Anomaly Index (SAI), and precipitation concentration Index (PCI).

**The coefficient of Variation (CV)** is a statistical measure that quantifies the temporal variability of hydroclimatic variables. It is computed by the ratio of standard deviation to the mean (Equation (8)) [36]. According to Mishra et al., the degree of variability in climate variables is classified under three categories based on CV values. When the (CV < 20 %), it is considered less variable; as the CV value lies in between (20 < CV < 30), it indicates moderate variability, while the CV value greater than 30 described high variability [36].

(8) $$CV=\frac{\sigma }{\mu }*100$$

Where CV is the coefficient of variation, σ is the standard deviation, and μ is the mean rainfall temperature and stream flow.

**Standardized Anomaly Index (SAI)** is a widely used index for climate change studies that measures the deviation in standard units between a data value and its mean. This index is used to compute the negative and positive anomalies of rainfall and temperature fluctuation in the basin [37]. Surendran et al. introduced different categories of SAI value ranges for identifying extreme rainfall events. SAI value of less than −1.65 indicates extreme drought, while severe drought occurs with values between −1.65 and −1.28. SAI values between −1.28 and −0.84 indicate moderate drought, while the rainfall is near the average, with SAI values of −0.84 to 0.84. Conditions are considered moderately wet if the SAI is between 0.84 and 1.28, very wet if between 1.28 and 1.65, and excessively moist with SAI values greater than 1.65 [38].

Regarding temperature, a warming period is considered to have occurred when the SAI value is above the long-term average. In contrast, a cooling period is indicated when it is below the long-term mean [39,40]. The standardized anomaly index is computed using an equation described (equation (9)).

(9) $$SAI=\frac{x-\mu }{\sigma }*100$$

Where SAI is the Standardized anomaly index of mean rainfall and temperature, x is the annual and seasonal rainfall or temperature value for a particular year, μ is the annual mean rainfall or temperature for observation, and σ is the seasonal and annual rainfall or temperature standard deviation for observation.

**Precipitation concentration index (PCI)**: PCI value for rainfall calculated to assess the rainfall distribution, and it is computed by the formula initially developed by Ref. [41] and later modified by Ref. [42] (Equation (10)).

(10) $$PCI=\frac{\sum_{i=1}^{12}P_{i}^{2}}{{\left(\sum_{i=1}^{12}P_{i}\right)}^{2}}$$

Where Pi is the rainfall amount for the month.

According to Oliver, PCI value can range from 8.3 % to 100 % [41]. Based on this classification, a PCI value of less than 10 indicates a uniform pattern of rainfall distribution (low precipitation concentration). The next category, PCI values between 11 and 15, denotes a moderate or mild precipitation concentration. PCI values from 16 to 20 indicate an irregular or seasonal distribution of the precipitation concentration. The last classification, with PCI values of 21 and above, shows extremely high precipitation concentration, characterizing situations with significant irregularity in precipitation concentration or where a large share of precipitation occurs over a limited period [41].

## 3 Results

### 3.1 Hydroclimate variable trend analysis

#### 3.1.1 Rainfall

The rainfall analysis results displayed a nonsignificant trend for most stations, including the sub-basin. The winter season rainfall showed a non-significant decreasing trend for more than 50 % of the stations’ and for the overall sub-basin. However, the other seasons' including annual rainfall, showed an increasing trend for most stations. Arjo station exhibited substantial upward trends in spring, with corresponding rates of +6.05 mm/year. Conversely, Gidayana station displayed a marginally non-significant negative trend, while the Agaro and Nolekaba stations showed significant positive trends in the summer. The Agaro, Arjo, and Nolekaba stations observed significant positive trends annually. The area-weighted average rainfall for the overall Didessa sub-basin showed an increasing trend for spring, summer, and annual scale, suggesting a rising trend in rainfall (Table 3).

**Table 3:** Summary trend analysis statistics for annual and seasonal rainfall.

Stations	Winter (DJF)		Spring (MAM)		Summer (JJAS)		Annual	
	Βsen	P- cal	Βsen	P- cal	Βsen	P- cal	Βsen	P- cal
Agaro	−0.16	0.849	1.57	0.24	5.55	**0.012∗**	5.61	**0.030∗**
Anger	−0.02	0.849	1.40	0.23	2.61	0.537	1.59	0.745
Arjo	0.26	0.694	6.05	**0.012∗**	5.38	0.058	13.98	**0.003∗∗**
Bedele	−0.14	0.812	2.22	0.438	2.27	0.319	6.41	0.071
Dembi	−0.86	0.212	−2.81	0.375	−2.88	0.329	−7.12	0.229
Didessa	0.02	0.888	2.13	0.110	2.01	0.410	5.32	0.229
Gatira	−0.44	0.435	1.04	0.567	1.37	0.172	1.36	**0.044**∗
Gidayana	0.09	0.786	0.89	0.099	−3.87	0.069	−1.33	0.711
Gimbi	0.14	0.386	2.36	0.061	−0.35	0.862	5.33	0.071
Nedjo	0.01	0.884	−1.22	0.465	1.43	0.481	1.67	0.580
Nekemte	−0.01	0.955	1.66	0.465	0.43	0.914	3.07	0.375
Nolekaba	−0.07	0.567	1.68	0.130	3.31	**0.013∗**	10.51	**0.003∗∗**
Shambu	−0.29	0.124	1.14	0.900	0.48	1.000	1.44	0.567
Sibusire	0.09	0.694	1.27	0.375	2.87	0.124	3.27	0.16

Didessa Sub-basin	−0.1	0.713	1.09	0.212	1.29	0.083	3.91	0.094

C βsen = Theil-Sen's slope and P- cal – calculated P-value, ∗, ∗∗ and ∗∗∗ significant at α = 0.05, α = 0.01 and α = 0.001 respectively.

#### 3.1.2 Minimum, maximum and mean temperature

The analysis results for annual minimum, maximum, and mean temperatures exhibited increasing trends for all stations and the overall sub-basin. However, Anger, Didessa, and Gatira stations showed a noteworthy negative trend, providing a comprehensive data view. A significant positive trend for the annual minimum temperature was observed at seven stations and for the overall sub-basin at the 0.01 %, 0.1 %, 1 %, and 5 % significance levels. Conversely, two stations exhibited a decreasing trend, with one significantly decreasing trend at the 5 % significance level. The annual maximum temperature displayed an increasing trend for Eleven stations and the overall mean of the sub-basin. Four stations and the overall sub-basin's results showed positive significant trends at the 0.0001, 0.001, 0.01, and 0.05 significance levels. The remaining three stations showed a non-significant decreasing trend (Table 4).

**Table 4:** Summary trend analysis statistics of annual minimum, maximum, and mean temperatures.

Stations	T_min		T_max		T_mean	
	Βsen	P- cal	Βsen	P- cal	Βsen	P- cal
Agaro	0.004	0.629	0.018	0.067	0.001	0.902
Anger	0.016	0.129	−0.009	0.502	0.015	0.086
Arjo	0.036	**0.0012∗∗**	0.067	**< 0.0001∗∗∗**	0.064	**< 0.0001∗∗∗**
Bedele	0.001	0.849	0.007	0.522	0.005	0.425
Dembi	−0.0007	0.955	0.038	**0.0002∗∗∗**	0.009	0.247
Didessa	0.013	0.1238	−0.010	0.276	0.013	0.078
Gatira	−0.017	**0.022∗**	−0.002	0.779	−0.009	0.242
Gidayana	0.023	**0.014∗**	0.003	0.831	0.013	**0.044∗**
Gimbi	0.001	0.090	0.007	0.286	0.007	0.196
Nedjo	0.006	0.680	0.015	**0.025∗**	0.010	0.204
Nekemte	0.019	**0.012∗**	0.035	**< 0.0001∗∗∗**	0.030	**< 0.0001∗∗∗**
Nolekaba	0.048	**0.0004∗∗∗**	0.006	0.412	0.034	**< 0.0001∗∗∗**
Shambu	0.058	**0.0006∗∗∗**	0.014	0.099	0.029	**< 0.0001∗∗∗**
Sibusire	0.048	**< 0.0001∗∗∗**	0.037	0.071	0.032	**0.0013∗∗**

Didessa Sub-basin	0.017	**0.0005∗∗∗**	0.027	**0.0023∗∗**	0.021	**< 0.0001∗∗∗**

T_min = Minimum Temperature, T_max = Maximum Temperature and T_Mean = Mean Temperature) βsen = Theil-Sen's slope and P- cal – calculated P-value, ∗, ∗∗ and ∗∗∗ significant at α = 0.05,α = 0.01, and α = 0.001, respectively.

The overall mean temperature followed similar patterns to the minimum and maximum temperatures for all stations and the sub-basin. The mean annual temperature showed an increasing trend for thirteen stations, with six stations significantly increasing. The sub-basin's overall maximum, minimum, and mean temperatures exhibited a rising trend, as described in Fig. 5.

#### 3.1.3 Stream flow

Analysis results for the annual and seasonal timescales showed a positive trend in the observed stream flow of all stations except Dembi and Big Anger (Table 5). The spring season demonstrated a significant rising trend for Arjo and Bunobedele at the 0.1 % and 5 % significance levels, respectively. At the same time, the winter, summer, and annual flow indicated a non-significant positive trend. Two distinct trends were observed in the upstream stations: Big Anger had a negative seasonal and annual time scale trend, while Little Anger showed a positive trend. Big Anger exhibited significant declining trends in the spring seasons at a 1 % significance level; Little Anger, on the other hand, showed statistically significant positive trends at 1 %, 5 %, and 5 % significance levels in the spring, summer, and annual levels, respectively.

**Table 5:** Summary trend analysis statistics for seasonal and annual stream flow.

Station	Winter (DJF)		Spring (MAM)		Summer (JJAS)		Annual	
	P- cal	Βsen	P- cal	Βsen	P- cal	Βsen	P- cal	Βsen
Arjo	0.757	0.060	**0.006∗∗**	0.533	0.223	1.0264	0.410	0.419
Bunobedele	0.291	0.010	**0.049∗**	0.015	0.245	0.131	0.446	0.049
Dembi	0.915	−0.003	1.000	−0.002	0.887	0.051	0.972	−0.013
Big Anger	0.283	0.51	**0.003∗∗**	−3.044	0.858	−2.26	0.371	−4.942
Little Anger	0.159	0.633	**0.002∗∗**	0.253	**0.013∗**	1.251	**0.0103∗**	0.464

### 3.2 Descriptive statistics and variability analysis of hydroclimate variables

#### 3.2.1 Seasonal and annual rainfall variability

**Fig. 3:**
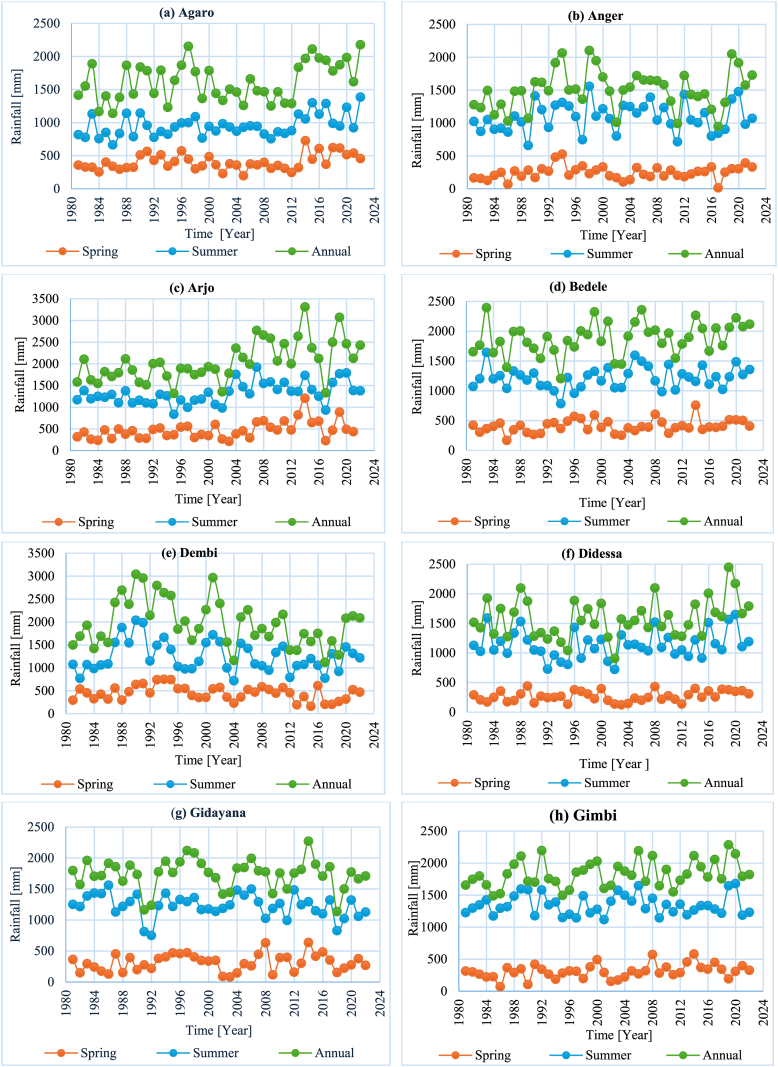

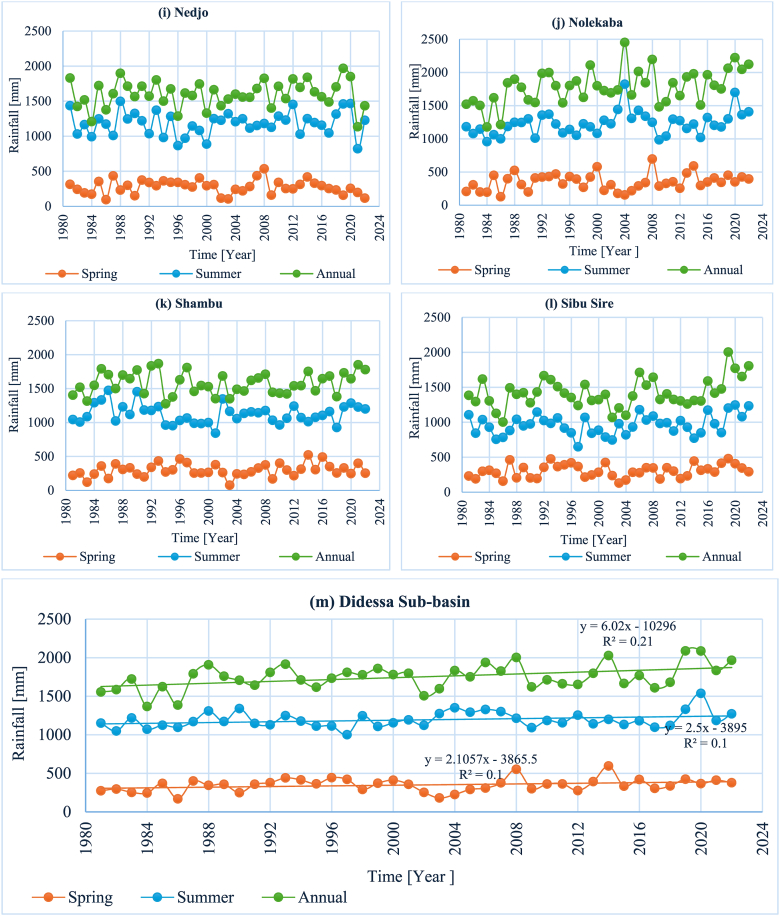
(a–m). Spatial and temporal average rainfall variabilities across the sub-basin and among a subset of stations in the Didessa Sub-basin.

**Coefficient of variability (CV):** The rainfall variability analysis result at the monthly timescale and winter season revealed high rainfall variability, with all stations having a CV > 30 %. Similarly, high rainfall variability was observed in the spring, with 11 stations having CV values greater than 30 % and three stations with CV values between 20 % and 30 % (Table 6). The overall sub-basin analysis result showed a CV value between 20 and 30, indicating moderate variability. The annual and summer seasons showed a similar pattern of rainfall variability, with approximately 79 % of the stations having CV values less than 20 % and the remaining stations having CV values between 20 % and 30 % (Table 6). Meanwhile, the overall sub-basin had a CV value for the summer season and annual rainfall data of less than 10 %. Additionally, Fig. 3 (a–m) graphically illustrates the rainfall variability of the sub-basin.

**Table 6:** Summary statistics of monthly, seasonal, and annual rainfall.

Station	Monthly		Winter		Spring		Summer		Annual	
	Mean (mm)	CV (%)	Mean (mm)	CV (%)	Mean (mm)	CV (%)	Mean (mm)	CV (%)	Mean (mm)	CV (%)
Agaro	135	67.1	90.8	61.9	403	29.5	953	17.3	1620	17.7
Anger	125	94.6	20.3	101.2	245	40.1	1089	20.0	1504	19.5
Arjo	171	80.0	58.7	63.6	474	42.3	1322	18.6	2046	22.2
Bedele	157	80.2	57.8	60.1	408	26.8	1216	14.8	1879	14.3
Dembi	166	76.3	84.2	80.8	453	35.3	1260	28.1	1993	27.4
Didessa	132	95.1	19.3	85.6	269	32.8	1139	20.3	1583	20.3
Gatira	161	67.4	94.5	48.5	460	23.4	1144	12.5	1928	12.2
Gidayana	145	92.5	26.4	81.5	312	44.0	1232	14.9	1738	13.7
Gimbi	153	97.9	16.9	98.8	308	34.9	1346	11.6	1832	11.2
Nedjo	134	98.9	9.7	98.3	276	35.6	1183	13.9	1601	11.6
Nekemte	169	90.9	35.0	75.8	379	35.4	1414	16.7	2023	17.3
Nolekaba	149	88.0	32.1	86.1	351	35.3	1230	14.2	1786	14.6
Shambu	132	92.6	39.8	72.5	299	31.5	1125	12.3	1579	10.3
Sibusire	119	86.6	32.4	55.9	305	30.5	964	14.8	1421	14.5

Didessa Sub-basin	146	86.4	40.9	49.1	349	24.5	1193	8.4	1749	9.3

##### 3.2.1.1 Standardized anomaly index (SAI)

**Fig. 4:**
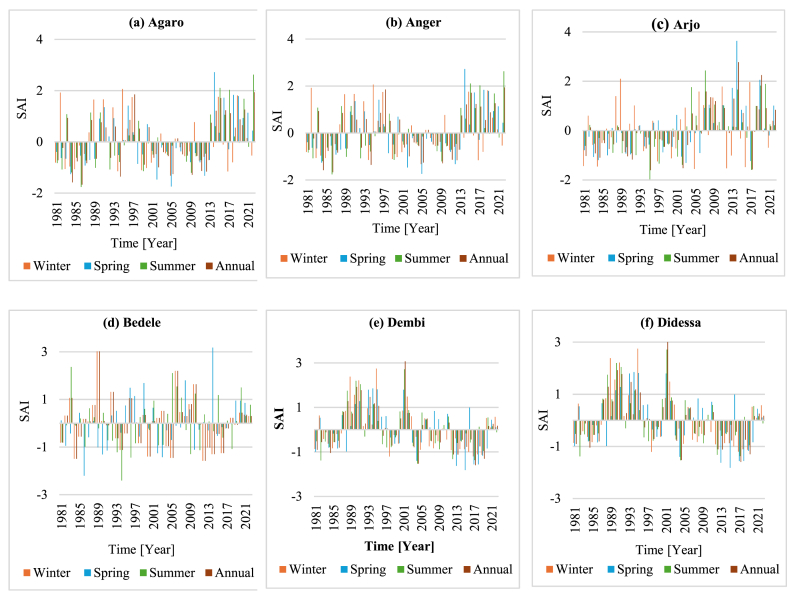

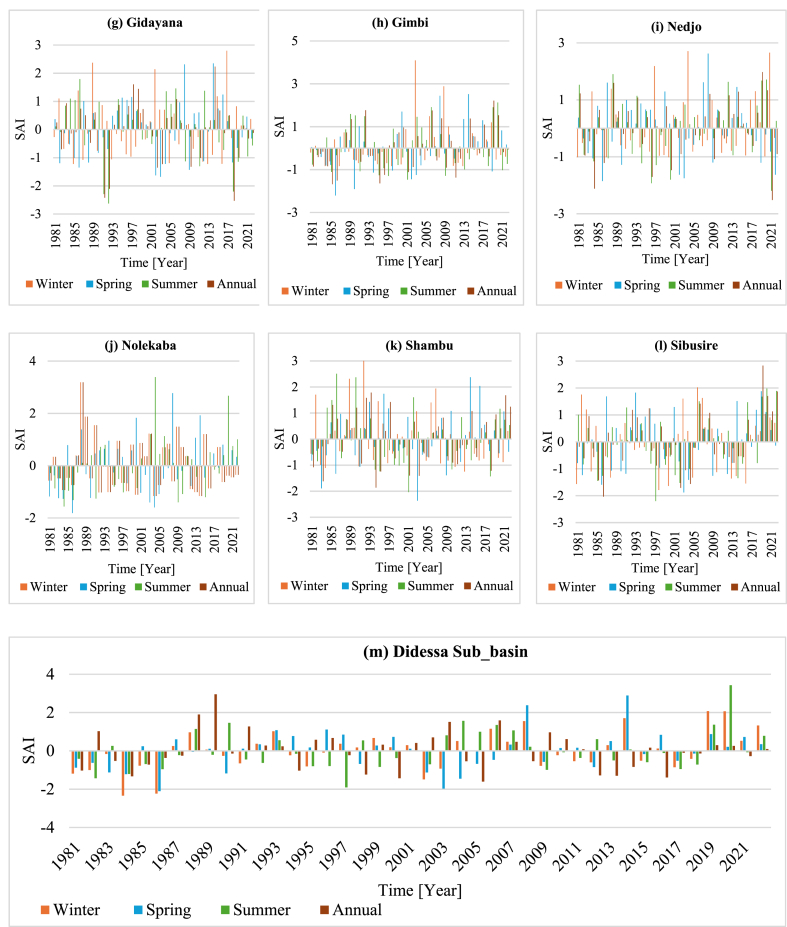
(a–m**)** shows the overall weighted average rainfall for the Didessa sub-basin (m) and the annual and seasonal standardized rainfall anomaly index for stations within the sub-basin.

The computed results on SAI at the annual and seasonal timescales indicated positive and negative values that characterize wet and dry years in all stations and the overall sub-basin (Fig. 4 a-m). The result noted that more than ten stations experienced extreme and severe drought years during the duration of all data, accounting for 2.28 %–11.9 % of all observations; the aggregate sub-basin analysis showed similar conditions. Conversely, SAI values for the moderate drought years ranged from 7.14 % to 16.67 % of all observations. Less than 7.14 % of the observations involved extreme or severe rainy conditions, whereas more than 73 % of the observed year showed normal rainfall conditions. The data indicate that 1984 was when the most severe drought occurred, with SAI values ranging from −2.53 to −1.66 (Fig. 4).

The result on the frequency, occurrence percentage, and severity of wet and dry episodes over 42 years prevailed normal rainfall across all sub-basin seasons, accounting for 60 %–64 % of the total observed period, indicating stable patterns for most of the time (Table 7).

**Table 7:** Occurrence of wet and dry periods per rainfall SAI over Didessa sub-basin.

Descriptions of SAI	Number of times in 42 years				Percentage of occurrence				Occurrence of events per 42 years			
	DJF	MAM	JJAS	Annual	DJF	MAM	JJAS	Annual	DJF	MAM	JJAS	Annual
Extreme dry	2	2	1	–	4.8	4.8	2.4	–	21	21	42	–
Severely dry	1	1	1	6	2.4	2.4	2.4	14.3	42	42	42	7
Mild dry	4	6	5	4	9.5	14.3	12.0	9.5	11.5	7	8.4	8.4
Normal	27	27	27	25	64.3	64.3	64.3	59.3	1.6	1.6	1.6	1.7
Mild wet	3	–	3	3	7.1	–	7.1	7.1	14	–	14	14
Severely wet	2	4	4	2	4.8	9.5	9.5	4.8	21	11.5	11.5	21
Extreme wet	3	2	1	2	7.1	4.8	2.4	4.8	14	21	42	21

∗DJF (December to February), MAM (March to May), and JJAS (June to September).

**Precipitation concentration index**: The rainfall distribution during the summer season had a PCI value of less than 10 for all stations, ranging from 8.57 to 8.95 at the Nedjo and Didessa stations, respectively, having an overall sub-basin weighted average value of 8.45. In the case of winter, spring, and annual rainfall, the PCI value ranges from 13.83 to 20.14, 12.84 to 17.28, and 10.43 to 15.02 for the Gatira and Gimbi stations, respectively. The overall weighted average PCI values calculated for the Didessa sub-basin were 14.62, 12.84, and 11.76 for winter, spring, and annual rainfall, respectively (Table 8).

**Table 8:** The PCI values of stations in the Didessa sub-basin.

Stations	Winter	Spring	Summer	Annual
Agaro	14.57	13.25	8.95	10.18
Anger	17.39	16.88	8.85	14.03
Arjo	16.04	14.59	8.81	11.54
Bedele	15.03	14.42	8.70	11.79
Dembi	14.73	14.33	8.89	11.01
Didessa	18.10	16.75	8.95	14.53
Gatira	13.83	12.84	8.72	10.43
Gidayana	18.67	16.29	8.70	13.86
Gimbi	20.14	17.28	8.68	15.02
Nedjo	19.28	16.73	8.57	14.92
Nekemte	16.03	15.76	8.73	12.80
Nolekaba	16.17	15.42	8.61	12.70
Shambu	16.17	13.74	8.82	12.66
Sibusire	16.81	15.38	8.86	11.97

Didessa Sub-basin	14.62	12.54	8.45	11.76

#### 3.2.2 Monthly and annual temperature variability

**Coefficient of variation (CV)**: A minimum and maximum CV values for monthly minimum temperature were observed at Gatira and Nedjo stations, with values of 2.85 % and 8.6 %, respectively. For the annual time scale analysis, Bedele station had a minimum CV of 3.86 %, and the maximum CV value of 15.93 % was recorded at Dembi station. The overall sub-basin means CV values were 5.41 % and 4.21 % for the monthly and annual timescales, respectively (Table 9).

**Table 9:** Summary statistics of monthly and annual minimum and maximum temperatures.

Stations	T_min				T_max				T_mean			
	Monthly		Annual		Monthly		Annual		Monthly		Annual	
	Mean (^0^C)	CV (%)	Mean (^0^C)	CV (%)	Mean (^0^C)	CV (%)	Mean (^0^C)	CV (%)	Mean (^0^C)	CV (%)	Mean (^0^C)	CV (%)
Agaro	12.2	6.6	12.2	10.3	27.9	4.9	27.9	4.9	20.1	3.8	20.1	4.5
Anger	15.5	6.3	15.5	8.3	29.2	8.2	29.2	3.1	22.3	6.7	22.3	3.2
Arjo	11.9	4.0	11.9	7.1	22.1	10.0	22.1	9.2	17.0	7.8	17.0	7.5
Bedele	12.7	5.4	12.7	3.9	25.5	6.9	25.5	3.1	19.1	5.5	19.1	2.7
Dembi	11.5	5.4	11.5	15.9	26.5	4.6	26.5	3.5	19.0	3.3	19.0	6.0
Didessa	15.7	8.8	15.7	5.3	30.6	8.7	30.6	2.9	23.1	6.3	23.1	2.5
Gatira	11.6	2.9	11.6	6.6	22.7	5.8	22.7	3.8	17.1	4.4	17.1	3.6
Gidayana	13.3	6.0	13.3	9.5	25.0	9.3	25.0	3.3	19.2	7.4	19.2	3.8
Gimbi	13.8	5.9	13.8	7.8	26.5	9.6	26.5	1.9	20.2	7. 8	20.2	3.0
Nedjo	12.9	8.6	12.9	10.9	26.1	9.7	26.1	2.3	19.5	5.9	19.5	4.0
Nekemte	12.8	5.8	12.8	5.7	24.5	9.8	24.5	3.7	18.7	7.7	18.7	3.6
Nolekaba	13.7	5.5	13.7	5.9	25.6	8.3	25.6	3.6	19.6	7.1	19.6	3.6
Shambu	11.3	6.9	11.3	8.1	23.1	8.6	23.1	3.7	17.2	7.0	17.2	4.1
Sibusire	13.8	6.3	13.9	7.3	27.8	9.6	27.8	2.9	20.8	6.7	20.8	3.4

Didessa Sub-basin	13.0	5.4	13.0	4.2	25.9	8.1	25.9	1.9	19.5	6.2	19.5	2.3

**Fig. 5:**
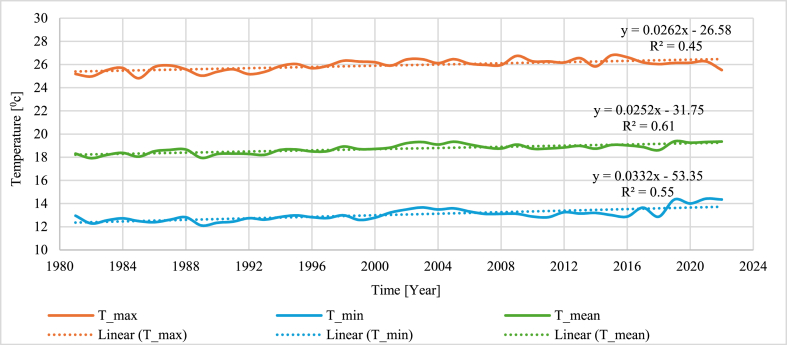
Maximum, minimum, and mean temperature variability of the Didessa sub-basin.

For the maximum monthly temperature, maximum CV values of 9.96 % and 9.18 % were observed at Arjo station at monthly and annual time scales, respectively. Meanwhile, the minimum CV value was recorded at Dembi and Gimbi stations on the monthly and annual scales. The overall sub-basin CV values of 8.09 % and 1.86 % were observed monthly and annually, respectively (Table 9). On the other hand, the mean temperature had a maximum CV value of 7.51 % at Arjo and a minimum value of 2.49 % at Didessa stations annually. For the monthly mean temperature, the maximum value was recorded at Dembi, and the minimum was observed at Gimbi station, with overall sub-basin CV values of 6.17 % and 2.26 %, respectively (Table 9). The overall temperature trend in the Didessa sub-basin exhibited an increasing trend (Fig. 5).

##### 3.2.2.1 Standardized anomaly index (SAI)

**Fig. 6(a–c):**
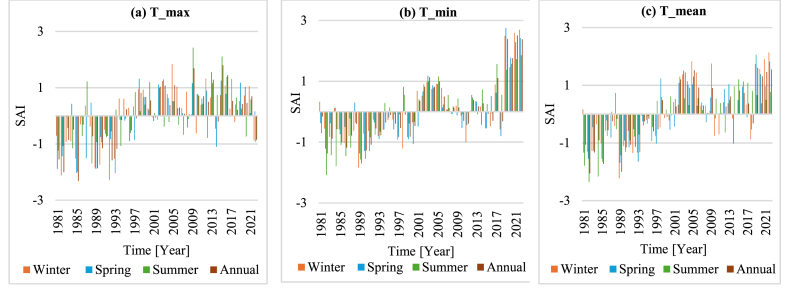
Seasonal and annual SAI for maximum, minimum, and mean temperature of the Didessa sub-basin.

The analysis of the seasonal and annual anomaly index results showed positive and negative values for maximum, minimum, and mean temperature data (Figure). 6(a-c)). The result showed the most negative anomalies in the 1980's, with temperature values lower than the baseline. Nonetheless, there was a shift towards positive anomalies in the late 1990s and early 2000s. The annual anomalies reflected seasonal trends, with negative values in the early 1980s and considerable positive anomalies in the 2000s. While there was a discernible trend towards rising temperatures in the 2000s, the 1990s were a transitional decade marked by various anomalies. This pattern continued in the 2010s and the first part of the 2020s, with primarily positive anomalies indicating increased temperatures throughout all seasons, especially in winter and spring (Fig. 5, Fig. 6(a–c)).

##### 3.2.2.2 Decadal temperature and precipitation change

**Fig. 7:**
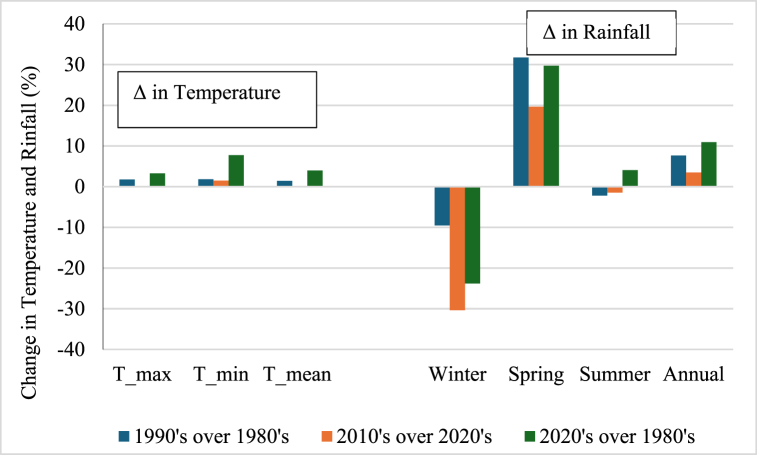
Decadal changes in temperature and rainfall.

The data show an increasing trend for temperature every decade. Moreover, winter rainfall is decreasing, especially between the 2010s and the 2020s, while spring, summer, and annual time scales are increasing (Fig. 7).

#### 3.2.3 Stream flow variability analysis result

The stream flow variability analysis result, based on the coefficient of variability, shows extremely high variability for the monthly, winter, and spring season data, with a CV value of greater than 30 %. For the summer season and annual stream flow data, the CV values lie between 20 % and 30 %, with the Dembi station having a CV value of less than 20 % and the upstream stations having CV values greater than 30 % (Table 10).

**Table 10:** Summarized statistical results of monthly, seasonal, and annual streamflow data.

Stations	Monthly		Winter		Spring		Summer		Annual	
	Mean (m^3^/s)	CV (%)	Mean (m^3^/s)	CV (%)	Mean (m^3^/s)	CV (%)	Mean (m^3^/s)	CV (%)	Mean (m^3^/s)	CV (%)
Arjo	107.88	106.11	21.27	41.45	20.03	61.03	226.62	24.82	106.88	24.10
Buno Bedene	11.62	115.76	1.51	32.62	1.14	68.31	26.24	20.68	11.62	24.28
Dembi	42.34	109.92	5.84	43.78	8.34	53.58	95.82	18.08	42.34	20.19
Big Anger	21.02	97.00	12.53	104.83	19.92	104.06	113.40	48.98	61.41	50.53
Little Anger	61.41	96.65	35.59	37.08	7.30	33.29	41.47	34.14	21.02	31.21

### 3.3 Rainfall patterns and their impact on stream flow

#### 3.3.1 Patterns of rainfall and stream flow

**Fig. 8:**
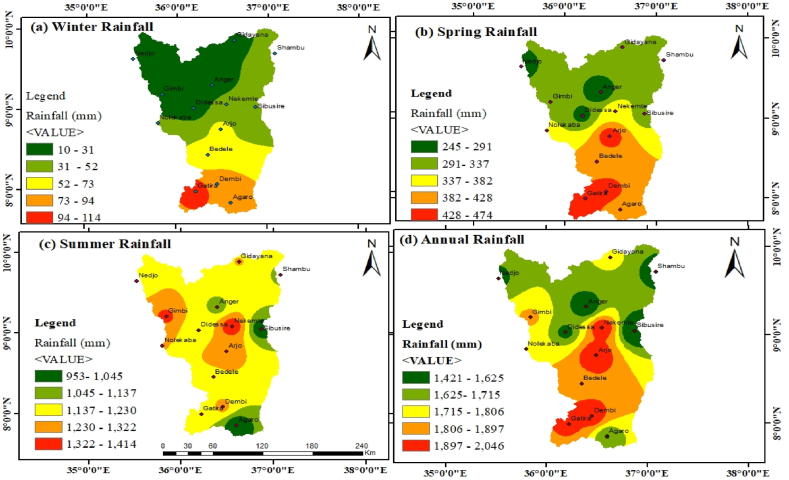
Mean seasonal and annual rainfall in the Didessa sub-basin.

The sub-basin experienced similar rainfall patterns for the annual and spring seasons, with most of the northern region having the second-lowest rainfall range of 291–332 mm in winter and 1625–1775 mm annually. In contrast, the sub-basin central, southwestern, and southern regions receive greater annual, winter, and spring rainfall. Most parts of the sub-basins perceive mid-range rain in the summer season with a value range of 1137–1230 mm. Meanwhile, the northwest portion of the sub-basin has the lowest rainfall during the winter. In general, 68 % of the annual rain occurs during the summer, with spring rainfall accounting for 20 % of the average annual rainfall (Fig. 8(a–d)).

**Fig. 9:**
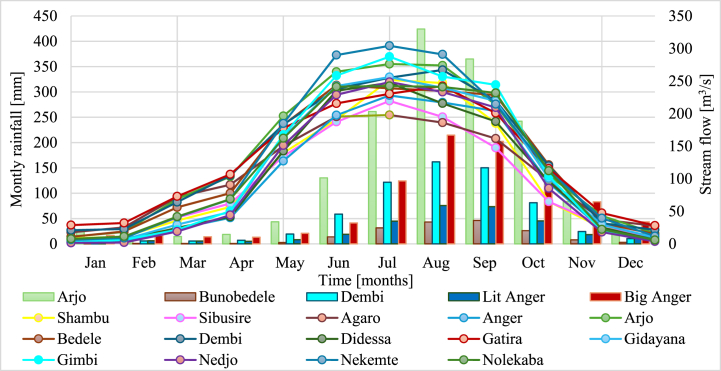
Monthly rainfall and stream flow patterns.

The mean monthly rainfall and stream flow data for the selected stations of the sub-basin showed a similar pattern. The maximum monthly rainfall and stream flow data for the Didessa sub-basin were observed during July and August, respectively. Rainfall increases from March to August and declines from September to February, with minimum concentrations observed between November and February. The overall weighted average rainfall data indicates that the sub-basin receives minimal rainfall of 11.1 mm in January and maximum rainfall of 318 mm in July. Similarly, the data on stream flow for the five gauged stations peaks in August after rising from May to August. The flow data declined from September to April, with the lowest flow recorded from February to April (Fig. 9).

#### 3.3.2 Correlation between rainfall and stream flow

**Fig. 10:**
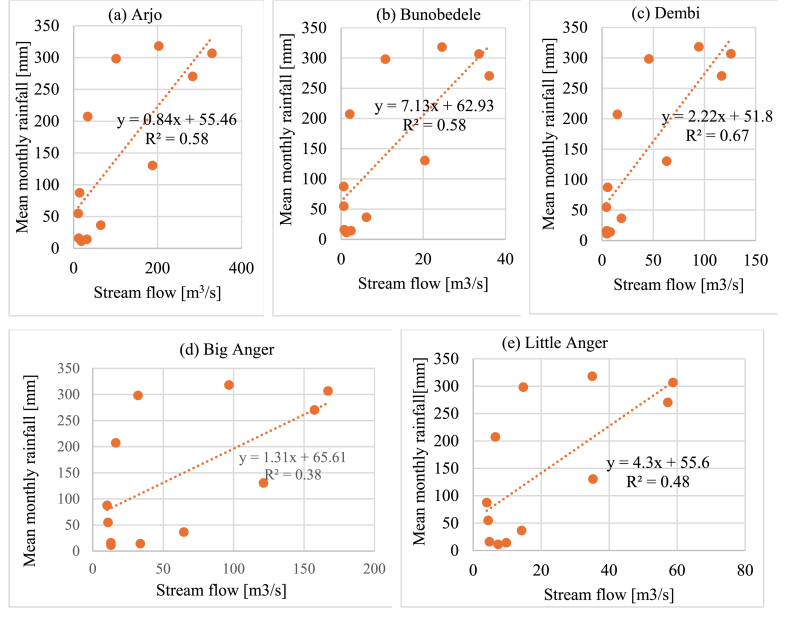
(a–e). Correlation between mean monthly rainfall and stream flow.

The correlation coefficient results revealed a positive correlation between rainfall and stream flow. The flow data at the Arjo, Bunobedele, and Dambi stations show a substantially higher degree of correlation than the upstream flow data, with correlation coefficient values of 0.58, 0.58, and 0.67, respectively. On the other hand, upstream stations showed a lower relation between rainfall and stream flow, with correlation coefficients of 0.38 and 0.48 for Big Anger and Little Anger, respectively (Fig. 10(a–e)). Also, the monthly and seasonal rainfall linkage with stream flow each month and on a seasonal basis was computed as illustrated in (Table 11). The strength of the correlation based on the correlation coefficient value classified as very high (0.7–1.0), high (0.5–0.7), moderate (0.3–0.5), low (0.1–0.3), and negligible (0.0–0.1) [43].

**Table 11:** Monthly and seasonal correlation between rainfall and stream flow.

Time scale	Correlation values of Gaged stations				
	Arjo	Dembi	Bunobedele	little Anger	Big Anger
Jan	0.1	0.2	0.1	−0.2	0.0
Feb	0.2	0.2	0.4	−0.3	−0.4
Mar	0.1	0.1	0.4	−0.1	0.1
Apr	0.4	0.7	0.4	0.7	0.5
May	0.6	0.6	0.5	0.4	0.4
Jun	0.4	0.4	0.4	−0.3	0.3
Jul	0.5	0.5	0.6	0.0	0.1
Aug	0.3	0.3	0.6	0.6	0.1
Sep	0.2	0.5	0.6	0.5	0.0
Oct	0.7	0.5	0.7	0.5	0.4
Nov	0.4	0.6	0.3	−0.1	0.6
Dec	0.2	0.1	0.5	−0.1	−0.5
Winter	0.1	−0.1	0.1	−0.2	−0.3
Spring	0.5	0.6	0.5	−0.1	0.3
Summer	0.5	0.5	0.7	0.4	0.2
Annual	0.6	0.5	0.6	0.5	0.4

∗Correlation very high (0.7–1.0), high (0.5–0.7), moderate (0.3–0.5), low (0.1–0.3), and negligible (0.0–0.1).

## 4 Discussion

The overall observed results on rainfall exhibited increasing trends in some stations while others showed decreasing trends at an annual and seasonal time scale. This may be attributed to the relative importance of the seasonal moisture sources and the orographic effects. Similar observations have also been reported; for instance, Mengistu et al. reported a non-significant trend of increased rainfall on annual and seasonal timescales for certain stations within the Abbay basin, indicating that the pattern of rainfall changes can vary significantly depending on the topography of the location [44]. Also, Tabari et al. & Dechassa et al. noticed non-uniform rainfall trends in various regions of Ethiopia [8,21]. Additionally, research by Samy et al. in the Upper Blue Nile basin revealed significant trends in rainfall patterns across different stations. Some parts of the basin showed significant increasing trends, while others experienced significant decreasing trends in annual and seasonal rainfall data [45].

The potential reasons for the variation of rainfall trends across different stations within the sub-basin's would be attributed to a complex interplay of factors. In which it would be due to natural variability in weather patterns, such as El Niño-Southern Oscillation (ENSO) or Indian Ocean Dipole (IOD), can significantly influence precipitation and temperature in different regions, including our study sub-basin. Long-term shifts in global climate, including rising temperatures and changing precipitation patterns, can also contribute to significant and non-significant trends in hydro-climatic variables. Geographic factors, such as elevation, topography, and land cover, can further influence local climate conditions in the sub-basin, affecting hydro-climatic variables. For example, higher elevations in the study area often experience colder temperatures and different precipitation patterns compared to lower elevations. At the same time, the land's slope, aspect, and orientation can affect wind patterns and solar radiation, potentially contributing to the variation.

The overall analysis results of CV values for the entire sub-basin were remarkably low for summer and annual rainfall per classification ranges of CV [36]. These low CV values underscore the stable rainfall patterns for both the summer and annually, suggesting that the Didessa sub-basin generally experiences steady rain during these periods, with only minor fluctuations. The findings are consistent with those of other researchers [20,22,29,46,47], who also observed that the annual and summer rainfall variability across different stations displayed similar patterns, generally characterized by low to moderate variability.

On the other hand, the monthly, spring, and winter rainfall data categorized under high rainfall variability range across most stations; this aligns with other studies [20,22,29,46,47]The overall average rainfall of the sub-basin experienced extreme rainfall variability in the monthly and winter timescale, while the spring season was moderately variable. These findings underscore the need for suitable adaptation strategies to support agriculture during the spring and winter. At the same time, the more consistent summer rainfall aids stable crop production during the main rainy season, highlighting the practical implications of the research for agricultural planning.

The SAI results for the sub-basin and stations in the sub-basin showed negative and positive values, where the negative values are considered drought years coinciding with or following El Nino events. The positive values were considered as wet years for the stations. The SAI values for the spring season had more negative values than annual and summer rainfall data, indicating the presence of more drought years during this season. Similarly, in the study by Tadese et al., higher negative SAI values were observed during the spring than in the annual and summer seasons [48]. These negative anomalies could be caused by shifting global climate patterns, the change in ocean temperature, or a human-induced factor that can alter the climate. This negative SAI value could imply agricultural productivity and water resource availability.

PCI offers important insights into precipitation distribution and concentration for efficient agricultural planning, water resource management, and climate adaptation methods. According to the study's findings, in the spring and winter seasons, most stations, including the sub-basin, show a moderate to irregular distribution of precipitation as per the classification range of PCI values [41]. The irregularity in rainfall during this season suggests the necessity of effective water management and adaptable farming methods to cope with variability. On the other hand, the sub-basin exhibits regular precipitation distribution during the summer, indicating the presence of consistent rainfall concentrations, which is supported by the findings of [45]. This season's regularity in rainfall concentration benefits agricultural and water resource management.

The temperature trend results analysis for the Didessa sub-basin experienced a substantially increasing trend with an average rising rate of 0.02 °C/yr. The rising temperature signals in the study area suggest that the sub-basin is highly vulnerable to global warming and needs action. The probable causes of rising temperatures in the sub-basin could be linked to increased greenhouse gas emissions from human activity, a worldwide contributor to climate change. Moreover, certain regional and local causes, such as deforestation and land use shifts from natural to agricultural or urban regions, can drastically increase temperature and alter natural ecosystems and climate patterns. Researchers from various parts of the world reported similar findings, concluding that there is an increasing trend in temperature due to increased greenhouse gas emissions and other local contributing factors [2,22,36,38,47,48].

The rise in temperature has implications for water resources and agriculture. It impacts agriculture and crop yields, straining irrigation systems and boosting pest and disease outbreaks. Temperature changes impact crop adaptability and productivity, while increased water demand and shortages might pose irrigation challenges. Warmer temperatures can also affect water quality by increasing pollutants, fostering algae blooms, diminishing natural purification, and causing water scarcity for agricultural and domestic use.

The findings on variability analysis showed a lower degree of temperature variability at both monthly and annual scales. These results are consistent with various studies conducted in different parts of Ethiopia, which also reported low-temperature variability across monthly, seasonal, and annual time scales [20,47]. The seasonal and annual maximum, minimum, and mean temperature anomaly index exhibit similar patterns. More negative values (wetter years) were recorded from 1981 to 1993, while more positive values indicative of warm years were observed from 2001 to 2022. The positive SAI value implies increased warming years during the latter period. Generally, the early 1980s had notable negative anomalies, especially in the spring of 1985 (−2.032) and 1993 (−2.040), along with the winters of 1982 (−2.117) and 1992 (−2.277). This pattern was also evident in the summer anomalies, which peaked in 1985 (−1.993) and 1988 (−1.692). Nonetheless, there was a shift towards positive anomalies in the late 1990s and early 2000s, especially during the winter, with peaks in 2003 (1.232), 2005 (1.841), and 2015 (0.736). A similar pattern was observed for spring anomalies, with 2013 recording the greatest value.

Thus, based on the results of SAI and the average change in temperature, the temperature has been increasing in recent decades over the preceding one across all decades, suggesting a warming trend that could potentially impact agriculture, water resources, and the regional climate. If not addressed, this warming trend could lead to significant changes in the regional environment, affecting not only agriculture and water resources but also the overall ecosystem of the region.

The result of the decadal precipitation change displayed decreased rainfall shifts during winter, which affects water availability and crops that rely on this rainfall. The positive changes in spring suggest shifts toward wetter conditions over the decades. This could benefit agriculture if planting seasons align with increased moisture availability. Still, it could also pose challenges related to excessive rainfall or waterlogging, potentially damaging crops and affecting soil quality. The overall rising annual rain, particularly when comparing the 2020s to the 1980s, suggests that the region has seen a general increase in rainfall over the long term. The increase in rainfall would have positive and negative implications for agriculture; it would have a probability of increasing water resources positively. However, it would affect agriculture by increasing leaching in the soil nutrients, creating delays in agriculture practices, and aggravating pests and diseases. Thus, applying possible agricultural and water management practices is crucial for the area.

The analysis of stream flow trends at various locations indicates significant seasonal and annual variations. At the downstream station, increasing trends were found at Arjo and Bunobedele, with significant increases during spring. At the same time, non-significant positive trends were identified at the winter, summer, and annual time scales. While the upstream stations revealed a mix of positive and negative trends, the Big Anger station showed extremely significant declining trends in spring, whereas no significant trends were observed in winter, summer, and annually. In Little Anger, a nonsignificant trend was observed in winter, while significant increasing trends appeared in spring, summer, and annually. This result also aligns with the findings of Duguma, who stated that the trend for Arjo stations was significantly increasing while the trend for Big Anger stations was substantially decreasing [49]. The findings exhibited severe stream flow variability at seasonal and monthly scales across all stream-gaged stations, particularly during the monthly, winter, and spring seasons. The downstream stations showed low to moderate rainfall variability during the summer and annually, but the upstream stations had higher flow variability with CV values greater than 30 %.

The mean monthly and seasonal rainfall data had heavy rainfall concentration between June and September, with July being the month with the highest rainfall across all stations. In contrast, the dry season, spanning from November to February, receives the least rainfall, with levels starting to increase in March. This rainfall pattern is mirrored in the monthly streamflow data, with the lowest streamflow observed from February to April, gradually rising in May.

Based on the rainfall and stream flow data patterns, the response of average stream flow lag to average rainfall over all stations was analyzed by comparing the annual cycle of this variable (Fig. 10). The lag period for rains over the stream flow is compared based on the month receiving maximum values on these variables along the annual cycle. According to the characteristics of these variables, maximum rainfall data for all stations occurred during July, while maximum stream flow data occurred during August after heavier rainfall in July. Thus, based on the result, the average stream flow had a one-month lag in response to the rainfall.

Rainfall and streamflow showed varying correlations throughout the year, with weak correlations during the dry months (January–March) and highly correlated during the rainy season (April–October), particularly at downstream stations like Arjo and Buno Bedene. This indicates a direct influence of rainfall on streamflow during wetter periods. Strong correlations in spring and summer underscore rainfall's significant contribution to streamflow, while winter correlations are weak or negative, reflecting a limited impact of rainfall. On an annual basis, moderate to high correlations (0.4–0.6) suggest that rainfall is a major driver of streamflow, particularly in downstream areas with more cumulative runoff. At the same time, the upstream stations display weaker or negative correlations, indicating that other factors play a role in streamflow variability.

Overall, positive correlations were observed across all stations, with downstream stations showing high correlations (values above 0.5), suggesting that streamflow is closely tied to rainfall variability in these areas. Conversely, upstream stations exhibit moderate correlations (values below 0.5), indicating that streamflow variability is influenced by factors other than rainfall. This analysis highlights the critical periods when rainfall drives streamflow, providing valuable insights for water management strategies to optimize water use and address seasonal variability in the Didessa sub-basin. Also, it suggests that rainfall is not the only factor that causes high stream flow variability. Other factors like land use, land cover change, human activity, and intervention may contribute more to stream flow variability. Similar justifications were observed by different studies conducted in the Awash and Abbay river basins [15,48].

## 5 Implications of the study

The research's conclusions have a significant implication on the agriculture, ecosystems, and biodiversity of the Didessa sub-basin. Consistent rainfall patterns during summer foster vegetation growth and development, providing food and habitat for various species and preserving ecosystem health and agricultural productivity [50]. However, extreme variations in rainfall during the winter and spring can cause habitat instability, affecting species that depend on steady water availability and potentially reducing biodiversity [51]. Additionally, the sub-basin's agriculture is more vulnerable during these seasons due to significant fluctuations in rainfall. To mitigate the effects of this unpredictability, effective water management and adaptive farming techniques, such as crop rotation, diversification, and irrigation scheduling [52].

Variations in precipitation patterns also directly influence water availability in rivers and streams, affecting the health of riparian zones and wetlands, which are vital habitats for many species [53]. Drought periods can cause water shortages that impact aquatic and terrestrial ecosystems, as demonstrated by negative SAI values [54]. Conversely, rainy years may cause flooding, leading to ecological disruption. Effective management and conservation techniques are essential in the Didessa sub-basin to preserve ecosystem services and conserve biodiversity, highlighting the interdependence of climate, water availability, and ecosystem health [55].

Climate change, through increased temperature, has profound implications for the Didessa sub-basin. Rising temperature trends indicate a potential increase in plant heat stress, which can lead to changes in species distribution and a decline in crop production. Furthermore, it suggests that societies are becoming more vulnerable to global warming, necessitating the establishment of climate adaptation plans to address potential effects on water resources, agriculture, and overall ecosystem health [14].

Variations in temperature and rainfall can impact the availability of resources, including food, raw materials, and water, affecting both wildlife and human societies that rely on them [56]. Ecosystems are crucial in regulating disease, water quality, and climate. Changes in ecosystem health can make these services more vulnerable to climate change and other environmental stressors [57]. Thus, the findings emphasize the importance of implementing adaptive management strategies to conserve ecosystems and biodiversity. This includes monitoring temperature and water availability fluctuations and conducting conservation measures for the benefit of vulnerable species and habitats [58]. The restoration of degraded ecosystems can assist in mitigating some of the harmful consequences of climatic variability. Efforts to restore riparian zones, wetlands, and other vital ecosystems can enhance ecosystem resilience [59].

The significant fluctuations in streamflow, especially in upstream regions, highlight the importance of implementing appropriate water resource management strategies. This includes constructing reservoirs and implementing water conservation measures to maintain a stable water supply yearly [60]. Additionally, these fluctuations can harm fisheries, aquatic plants, and other organisms that require steady water conditions [61]. There is a positive association between streamflow and rainfall, highlighting the significance of precipitation in maintaining water supplies for both ecological and human needs [53]. Adaptive management solutions are crucial for addressing the effects of climatic variability on water availability and ensuring sustainable water resource management in the sub-basin [62].

The findings from the study on the Didessa sub-basin hydroclimate trends and variability can lead to specific recommendations for water resource management by highlighting areas of concern and potential strategies. For instance, the observed increasing rainfall trends and significant temperature rise suggest enhanced water storage and conservation measures to manage the increased water availability and mitigate potential flooding. The mixed trends in stream flow data indicate that tailored strategies are necessary for different parts of the sub-basin; upstream areas with declining flow might benefit from reforestation and soil conservation efforts to enhance water retention, while downstream areas with increasing flow could require improved infrastructure to handle higher water volumes. By understanding these trends, policymakers and water resource managers can develop actionable strategies such as constructing new reservoirs, implementing efficient irrigation practices, and promoting sustainable land use to ensure the long-term sustainability of water resources in the Didessa sub-basin.

## 6 Conclusions

This study examined the trends and variability of hydroclimate variables in the Didessa sub-basin, focusing on rainfall, temperature, and streamflow patterns and their interrelationships. The study employed Modified Mann-Kendall, CV, SAI, and PCI analysis methods. The findings underscore the importance of understanding these trends, as they have a significant impact on water resource management. The rising trends in temperature and rainfall, for instance, suggest a shift toward increased rainfall and warming in temperature, which can inform future management strategies. From the results of the streamflow analysis, it is concluded that the downstream flow had a significant increasing trend while the upstream displayed a significant decreasing trend. This implied that, unlike the upstream flow, the downstream flow directly correlates with the increment in precipitation. It suggests that other factors beyond rainfall can influence streamflow in these areas. These findings provide valuable insights into the trends and variability of hydroclimate variables in the Didessa sub-basin, which can inform future water resource management strategies for policymakers and water managers. However, the sub-basin's significant role within the larger Abbay basin makes it need additional monitoring stations for a more comprehensive understanding and studies at a sub-catchment level investigation on hydroclimate variables variability and its driving factors.

## CRediT authorship contribution statement

**Selamawit Bekele:** Writing – review & editing, Writing – original draft, Visualization, Validation, Software, Resources, Methodology, Investigation, Formal analysis, Data curation, Conceptualization. **Tena Alamirew:** Writing – review & editing, Validation, Supervision, Methodology, Conceptualization. **Sintayehu Fetene Demessie:** Writing – review & editing, Visualization, Validation, Methodology, Data curation.

## Data availability statement

Data will be made available on request.

## Declaration of competing interest

The authors declare that they have no known competing financial interests or personal relationships that could have appeared to influence the work reported in this paper.
